# A high‐throughput microfluidic mechanoporation platform to enable intracellular delivery of cyclic peptides in cell‐based assays

**DOI:** 10.1002/btm2.10542

**Published:** 2023-05-13

**Authors:** Stephen H. Kasper, Stephanie Otten, Brian Squadroni, Cionna Orr‐Terry, Yi Kuang, Lily Mussallem, Lan Ge, Lin Yan, Srinivasaraghavan Kannan, Chandra S. Verma, Christopher J. Brown, Charles W. Johannes, David P. Lane, Arun Chandramohan, Anthony W. Partridge, Lee R. Roberts, Hubert Josien, Alex G. Therien, Erik C. Hett, Bonnie J. Howell, Andrea Peier, Xi Ai, Jason Cassaday

**Affiliations:** ^1^ Merck & Co., Inc. Cambridge Massachusetts USA; ^2^ Merck & Co., Inc. West Point Pennsylvania USA; ^3^ Merck & Co., Inc. Kenilworth New Jersey USA; ^4^ Agency for Science, Technology and Research (A*STAR) Singapore Singapore; ^5^ MSD International Singapore Singapore

**Keywords:** automation, cell‐based assays, cyclic peptides, intracellular delivery, microfluidics, protein–protein interactions

## Abstract

Cyclic peptides are poised to target historically difficult to drug intracellular protein–protein interactions, however, their general cell impermeability poses a challenge for characterizing function. Recent advances in microfluidics have enabled permeabilization of the cytoplasmic membrane by physical cell deformation (i.e., mechanoporation), resulting in intracellular delivery of impermeable macromolecules in vector‐ and electrophoretic‐free approaches. However, the number of payloads (e.g., peptides) and/or concentrations delivered via microfluidic mechanoporation is limited by having to pre‐mix cells and payloads, a manually intensive process. In this work, we show that cells are momentarily permeable (*t*_1/2_ = 1.1–2.8 min) after microfluidic vortex shedding (μVS) and that lower molecular weight macromolecules can be cytosolically delivered upon immediate exposure after cells are processed/permeabilized. To increase the ability to screen peptides, we built a system, dispensing‐microfluidic vortex shedding (DμVS), that integrates a μVS chip with inline microplate‐based dispensing. To do so, we synced an electronic pressure regulator, flow sensor, on/off dispense valve, and an x‐y motion platform in a software‐driven feedback loop. Using this system, we were able to deliver low microliter‐scale volumes of transiently mechanoporated cells to hundreds of wells on microtiter plates in just several minutes (e.g., 96‐well plate filled in <2.5 min). We validated the delivery of an impermeable peptide directed at MDM2, a negative regulator of the tumor suppressor p53, using a click chemistry‐ and NanoBRET‐based cell permeability assay in 96‐well format, with robust delivery across the full plate. Furthermore, we demonstrated that DμVS could be used to identify functional, low micromolar, cellular activity of otherwise cell‐inactive MDM2‐binding peptides using a p53 reporter cell assay in 96‐ and 384‐well format. Overall, DμVS can be combined with downstream cell assays to investigate intracellular target engagement in a high‐throughput manner, both for improving structure–activity relationship efforts and for early proof‐of‐biology of non‐optimized peptide (or potentially other macromolecular) tools.

## 1 INTRODUCTION

Enabling intracellular delivery (ICD) of impermeable molecules has the potential to facilitate and accelerate multiple aspects of biological research, yet it presents with important challenges. ICD has historically been achieved through multiple approaches,^1^, ^2^ including electroporation, vector‐mediated transfer, and mechanoporation, the process of transferring molecules into cells using mechanical energy. Recent advances in microfluidic methods have enabled novel mechanoporation approaches, including nanoneedle penetration, micro/nanoinjection, cell squeezing, cell stretching, and fluid shearing.^3^ These methods have shown the ability to efficiently deliver various cargos into a wide range of cells, often with minimal cell perturbation. However, the low throughput of these techniques, primarily due to the requirement for pre‐mixing cells with the cargo of interest prior to microfluidic processing, has been a significant bottleneck toward leveraging these techniques in cell‐based screens supporting drug discovery efforts.

Cyclic peptides are a compelling modality to target notoriously difficult‐to‐drug intracellular protein–protein interactions (PPIs), due to their size and ability to bind flat PPI interfaces with exceptional affinity and selectivity.^4^, ^5^ These peptides, which are generated through structure‐based design or via screening combinatorial libraries against a target of interest, typically lack cell permeability due to their size and hydrophilicity.^6^, ^7^, ^8^ Therefore, there is often a disconnect in potency between biochemical assays and cell‐based assays, making the discovery of cyclic peptides for intracellular PPIs a major challenge.^9^ Indeed, out of 18 cyclic peptides approved for clinical use in the past two decades—only two, romidepsin and voclosporin, modulate intracellular targets.^10^

Microfluidic‐based mechanoporation and subsequent ICD can be a useful approach to study intracellular target engagement of cyclic peptides without the use of exogenous transfection reagents or electrical fields. Microfluidic vortex shedding (μVS) is a fluid shearing‐based technique that was recently demonstrated to be effective at delivering mRNA and Cas9 ribonucleoprotein into primary human T cells.^11^, ^12^ μVS is induced when fluid flows past micron‐scale posts (40 μm diameter with 20 μm gaps) to create turbulent oscillating flow motion, which disrupts the lipid membrane.^11^, ^13^ Advantages of μVS over other microfluidic ICD approaches are the high flow rate and reduced clogging events, which are a function of the geometry that accompany the desired cell‐permeabilizing hydrodynamic conditions.

While μVS is being developed to enable cell engineering for cell therapy‐based applications, we envisioned that this method (and its inherent advantages mentioned above) could also be exploited to better understand target engagement of cell‐impermeable molecules in high‐throughput cell‐based assays. To improve on the existing throughput of μVS methodologies, we integrated a μVS chip with inline microplate‐based dispensing, in a system we call dispensing‐microfluidic vortex shedding (DμVS). We designed and constructed the DμVS system to rapidly shuttle permeabilized cells to standard microplates containing various peptides at different concentrations. We then validated the DμVS method using p53/MDM2 peptides in both a click‐chemistry‐based permeability assay (NanoClick, 96‐well format), as well as a functional p53 reporter assay (96‐ and 384‐well format). This combination of vector‐free ICD, microfluidics, and high‐throughput‐amenable cell‐based assays, could have a broad impact in drug discovery, enabling the development of novel modalities against challenging intracellular targets.

## 2 MATERIALS AND METHODS

### 2.1 Delivery of fluorescently labeled 3 kDa dextran into HeLa and HCT116 cells

The human cell lines HeLa and HCT116 were purchased from American Type Culture Collection (ATCC, Manassas, VA). HeLa cells were maintained in Dulbecco's Modified Eagle Medium (10569‐010, Gibco) with 10% fetal bovine serum (FBS, Gibco 10082‐139), and antibiotics (penicillin [100 units/ml] and streptomycin [100 μg/ml], Gibco 15140‐122) in 5% CO2 at 37°C. HCT116 cells were maintained in McCoy's 5A Medium (ATCC, 30–2007) with 10% FBS, and antibiotics (penicillin [100 units/ml] and streptomycin [100 μg/ml]), in 5% CO_2_ at 37°C. AlexaFluor‐488 dextran (AF488‐dextran, 3 kDa MW) was purchased from Invitrogen (D34682).

AF488‐dextran was delivered to cells using the Hydropore™ microfluidic vortex shedding (μVS) system with 40 μm inline‐filter devices from Indee Labs (Berkeley, CA).^11^ Cells (HeLa or HCT116) were suspended at 3 × 10^6^ cells/ml in 0.2‐μm filtered PBS with 3% FBS (v/v) in 1.5 ml Eppendorf Biopur tubes (05‐402‐24B, Fisher Scientific). AF‐488‐dextran was added at 0.2 mg/ml at a final volume of 0.5 ml. For “post‐mixed” samples, 0.45 ml of cells in buffer were in the Eppendorf tube and 50 ul of AF‐488‐dextran (or buffer vehicle) were added to the bottom of 15 ml conical collection tubes. Eppendorf tubes were connected to μVS system with an Elveflow microfluidic reservoir adaptor (LVF‐KPT‐XS‐2_2, Darwin Microfluidics, France). Samples were processed at 120 psi and received in the collection tube. After μVS processing, cells were pelleted by centrifugation (300× g, 5 min), and the buffer with excess AF‐488‐dextran was removed and discarded. Media with FBS was added and the cells were incubated at 37°C, 5% CO_2_ for 1.5 h. After incubation, cells were washed, labeled with LIVE/DEAD™ Fixable Yellow Dead Cell Stain Kit (L34959, Invitrogen) according to the manufacturer's protocol, and analyzed via flow cytometry.

For the kinetic analysis of membrane resealing, the zero time point samples were processed as described above for “post‐mixed” samples. For subsequent time points, the suspension was passed through the μVS system in bulk from a 15 ml Falcon tube with P‐CAP metal reservoir cap (Fluigent, MA) and added to AF488‐dextran at pre‐determined timepoints (1, 2, 4, 8, 16, and 32 min). Cells were then processed and analyzed as described above. Data represent mean and standard deviation among at least two replicates. Data were plotted in GraphPad Prism. A one‐way ANOVA was performed to compare the effect of μVS processing on AF488‐dextran delivery, followed by Sidak's multiple comparisons test only on AF488‐dextran treated samples. A one phase decay model was used to fit a curve to the kinetic data.

### 2.2 Setup and characterization of DμVS system

An N_2_ tank (N5.0, cylinder size 300, Middlesex Gases, MA) supplies air through 1/8” OD FEP tubing to an electronic pressure regulator (VEAA‐L‐3‐D11‐Q4‐V1‐1R1, Festo). Using 0‐10 VDC command, the proportional‐pressure regulator provides indicated head‐pressure to a 15 mL Falcon tube with P‐CAP metal reservoir cap. Liquid is flowed from the reservoir tube through 1.5 mm OD PTFE tubing through a liquid flow sensor (SLF3S‐1300F, Sensirion AG, Switzerland) to the Hydropore™ μVS system containing a 40 μm inline‐filter device. Upon leaving the device, fluid/cell suspension travels to a high‐speed 2‐way axial flow microsolenoid valve (INKX0514300A A, Lee Company) and exits through a 0.01” ID dispense nozzle (INZA5100914K A, Lee Company) onto any microplate of standard SBS footprint of the user's choosing, including custom grid labware that can also be defined with a maximum density of 384 positions. The microplate is situated in a 3D‐printed holder, which is stationed on a planar surface gantry (EXCM‐30, Festo). The dispense valve and tip are mounted onto a spindle axis (ELGC‐BS‐KF‐45‐200‐10P, Festo), which lowers the tip in the z‐direction to a configurable height above the microplate when the DμVS program is activated.

The DμVS process is coordinated by an automation software, Telios, which was developed in‐house using LabVIEW programming (National Instruments, Austin, TX).^14^, ^15^ The user writes a plate map in a .csv file, indicating the plate layout and desired volume filled per well. Once uploaded, the user can select the desired pressure for the system to run at and system prime volume (optional). The user would load a new μVS device into the chip housing unit, put desired microplate onto x‐y stage, and connect a cell suspension (or cell‐free solution) to the P‐CAP. Once these are ready the user can initiate the DμVS program. The software sends a command to close the dispense valve, followed by a command to the pressure regulator to initiate pressure. Next, Telios sends a command to the x‐y stage to move the plate to the desired location relative to the dispense tip and to lower the dispense tip to the configured height (typically a few millimeters above microplate). Once the dispense tip and plate are in position, a command is sent to open the valve. Due to the applied pressure, cells (or solution) are pneumatically pushed through the system and the flow sensor sends a running count of the displaced volume in the DμVS system every 20 ms. Once the target volume is achieved, Telios commands the valve to close, followed by a command to move the plate to the next desired location, and then another command to re‐open the valve. This process is repeated until the uploaded plate map is completed. At completion, the valve is closed, dispense tip is returned to resting z‐height, and the filled microplate is returned to the home position.

μVS devices are discarded after every plate. The dead volume of the downstream tubing and parts of the μVS device is ~200 μl. Therefore, this is the volume that must be displaced before mechanoporated cells are delivered to their respective payload. While the flow rate of the DμVS system at 120 psi is ~8 ml/min, this is frequently interrupted by the valve closing for the time that it takes to move a plate. Therefore, the residence time that it takes for cells to clear the dead volume is dependent on flow rate, volume dispensed per well, and plate format (move time between wells). Within this work, the residence time ranges from ~2.8 s (dispensing 100 μl on 96‐well plate) to as long as ~8.5 s (dispensing 20 μl on 384‐well plate).

For characterization of uniformity of dispensing volumetric fluid, fluorescein isothiocyanate‐dextran (average molecular weight 20,000; FD20, Sigma‐Aldrich) was used as a fluorescent tracer. FD20 was solubilized at 0.1 mg/ml in PBS and 0.2 μm‐filtered. A reservoir of FD20 was connected to the DμVS system and processed at 120 psi. The dispense volume was set at 50 μl for a 96‐well plate (Costar 3915) and 20 μl for a 384‐well plate (Corning 3764). An equivalent volume of PBS was pre‐filled on the plate to mimic the conditions at which the cell‐based assays described below were performed. For characterizing uniformity of cell suspension dispensing, HeLa cells were resuspended in 0.2 μm‐filtered PBS at 200,000 cells/ml and labeled with 10 μM CellTracker™ Red CMTPX Dye (C34552, Invitrogen). Similar to the volumetric characterization, cells were DμVS‐processed at 120 psi and dispensed at 50 or 20 μl (for 96‐ and 384‐well plates, respectively) into wells that were pre‐filled with an equivalent volume of PBS. All wells were filled with FD20 or CellTracker‐labeled HeLa cells except for three wells on a 96‐well plate and six wells on a 384‐well plate, to serve as background controls. After dispensing, fluorescence was measured on a PHERAstar FSX microplate reader (BMG Labtech).

### 2.3 NanoClick permeability assay

NanoBret 618‐azide (NB618Az), DIBAC‐chloroalkane (DIBAC‐CA), and Intracellular TE NanoGlo Substrate/Inhibitor (N2160) were purchased from Promega. Assay‐ready frozen (ARF) HeLa NanoClick cells were prepared as previously described.^16^ Cells were thawed, resuspended in Opti‐MEM (11058‐021, Invitrogen) with 1% v/v FBS, and allowed to incubate overnight on 150 mm tissue culture plates (Corning 430599). Peptides were synthesized as previously described.^16^, ^17^ To set up the assay, peptides were pre‐plated in 50 μl Opti‐MEM on opaque white 96‐well plates (Corning 3917) using a HP D300E digital dispenser. Maximum DMSO concentration used was 0.3% v/v. After overnight incubation, media was removed and replaced with 3 μM DIBAC‐CA in Opti‐MEM with 1% v/v FBS. Cells were incubated for 1 h with DIBAC‐CA at 37°C, then washed twice with PBS and treated with 0.05% v/v trypsin to lift cells. Cells were spun down for 5 min at 300× g, resuspended in Opti‐MEM (without FBS), 70‐μm strained (130‐098‐462, Miltenyi Biotec) and then brought to a final cell density of 2 × 10^5^ cells/ml in PBS. Cell suspension was connected to the 15 ml P‐CAP of the DμVS system, and 2 ml were used to prime the system. DμVS process was then initiated at 120 psi and 50 μl were dispensed to microplate containing Opti‐MEM with peptides according to predefined plate map in the Telios software. Unprocessed cells and cells treated with digitonin (50 μg/mL; Sigma‐Aldrich D141) were added to separate 96‐well plates in parallel. After DμVS processing/mixing with peptide, cells were spun down for 1 min at 100× g and then incubated at 37°C with 5% CO_2_ for 4 h. NB618Az was diluted to 40 μM in Opti‐MEM, and 33 μl was added to each well, yielding a final concentration of 10 μM. After 1 h incubation (37°C, 5% CO_2_), 50 μl of a NanoBRET Nano‐Glo Substrate and extracellular NanoLuc Inhibitor solution (6 μl substrate, 2 μl inhibitor per 1 ml of Opti‐MEM) was added to each well, and plate was read on a PHERAstar FSX microplate reader (BMG Labtech) immediately. NanoBRET ratio was calculated using the sample value (S) and the average of the vehicle controls for each respective processing condition (mean_veh_):

$$\text{NanoBRET ratio}=\frac{S}{{\text{mean}}_{\mathrm{veh}}}$$

EC_50_ values were calculated using the three parameter inhibitor versus response method in GraphPad Prism. Dose response experiments were performed with triplicates on plate and repeated at least three separate times. The plate‐wide single concentration experiments with 32 on‐plate replicates were performed twice independently. A one‐way ANOVA was performed to compare the effect of peptide addition under both unprocessed and DμVS‐processing conditions, followed by Dunnett's multiple comparisons test comparing peptide addition to vehicle‐treated samples.

### 2.4 HCT116 p53 β‐lactamase reporter gene functional assay

The CellSensor™ p53RE‐*bla* HCT116 cell line (K1640, Thermo Scientific) was used for functional characterization of p53 pathway activation by DμVS‐delivered peptides. Assay was performed similarly to how previously described.^17^ These cells harbor a stable integration of a β‐lactamase reporter gene under control of the p53 response element. Cells were maintained in McCoy's 5A medium (16600‐082, Invitrogen) with 10% dialyzed FBS (26400‐036, Invitrogen), blasticidin (R210‐01, Invitrogen), and penicillin/streptomycin according to the manufacturer's protocol. Peptides were synthesized as previously described.^16^, ^17^ To set up the assay, peptides were first pre‐plated in assay medium (Opti‐MEM supplemented with 0.1 mM non‐essential amino acids [11140‐050, Invitrogen], 1 mM sodium pyruvate [11360‐070, Invitrogen], and penicillin/streptomycin) without FBS on 384‐well plates (Corning 3764) or 96‐well plates (Corning 3603) using a HP D300E digital dispenser (20 μl 384‐well, 50 μl 96‐well). Maximum DMSO concentration used was 0.3% v/v. Cells were washed, resuspended with 0.05% v/v trypsin, spun down for 5 min at 300× g, and resuspended in PBS. Cells were 70‐μm strained and then brought to a final cell density of 3 × 10^6^ cells/ml in PBS. Cell suspension was connected to 15 mL P‐CAP of the DμVS system, and 2 mL were used to prime the system. DμVS process was then initiated at 120 psi and 20 μL (384‐well) or 50 μL (96‐well) were dispensed to microplate in wells that were pre‐defined in the Telios software and pre‐loaded with peptides 2× in assay medium. Unprocessed cells were added to the microplate in parallel. After DμVS processing/mixing with peptide, cells were incubated at 37°C for 4 h. β‐lactamase activity was detected using the ToxBLAzer loading kit (K1095, Invitrogen) according to the manufacturer's protocol. Plates were measured using the PHERAstar FSX microplate reader with a LiveBlazer optic module (1400‐G3M‐FI‐410‐530‐460, BMG Labtech). Maximum p53 activity was defined as the β‐lactamase activity induced by 30 μM MP‐081 (mean_pos_). Percent activation of individual samples (*S*) was calculated using the following equation, where mean_veh_ represents the mean of vehicle controls:

$$\%\;\text{activation}=\frac{S-{\text{mean}}_{\mathrm{veh}}}{{\text{mean}}_{\mathrm{pos}}-{\text{mean}}_{\mathrm{veh}}}\times 100$$

*Z*‐factors were calculated using the formula:

$$Z^{\prime }=1-\frac{3\sigma_{\mathrm{pos}}+3\sigma_{\mathrm{veh}}}{\left|{\text{mean}}_{\mathrm{pos}}-{\text{mean}}_{\mathrm{veh}}\right|}$$

Mean_pos_ and *σ*_pos_ represent the mean and standard deviation, respectively, of the raw ratiometric values for 30 μM MP‐081 and mean_veh_ and *σ*_veh_ represent the mean and standard deviation of the vehicle controls. Dose–response data EC_50_ values were determined using the “[Agonist] versus response—Variable slope (four parameters)” algorithm in GraphPad Prism. Single concentration data for expanded peptide set were analyzed using unpaired *T*‐tests with false discovery rate (FDR, 1.00%) multiple comparisons correction using the two‐stage step‐up (Benjamini, Krieger, and Yekutieli) method. Data were accumulated from at least five independent experimental runs.

### 2.5 MDM2 binding assay

MDM2 protein generation and binding studies were performed as described.^17^ Briefly, binding was performed using MDM2 (1–125) protein titrated against 50 nM carboxyfluorescein (FAM)—labeled 12/1 peptide13 (FAM‐RFMDYWEGL‐NH2). The apparent *K*_D_ value of FAM‐labeled 12/1peptide was determined to be 13.0 nM and subsequently used to determine the apparent *K*_D_ values of the respective competing ligands in competition assays in fluorescence anisotropy experiments. Titrations were carried out with the concentration of MDM2 held constant at 250 nM and the labeled peptide at 50 nM. The competing molecules were then titrated against the complex of the FAM‐labeled peptide and protein. Curve‐fitting was carried out using Prism 4.0 (GraphPad) to determine *K*_D_ values. Readings were carried out with an Envision Multilabel Reader (PerkinElmer). Experiments were carried out in PBS (2.7 mM KCl, 137 mM NaCl, 10 mM Na_2_HPO4, and 2 mM KH_2_PO_4_ [pH 7.4]) and 0.1% Tween 20 buffer. All titrations were carried out in triplicate.

## 3 RESULTS

### 3.1 μVS induces a short window of cell permeability after processing

**FIGURE 1:**
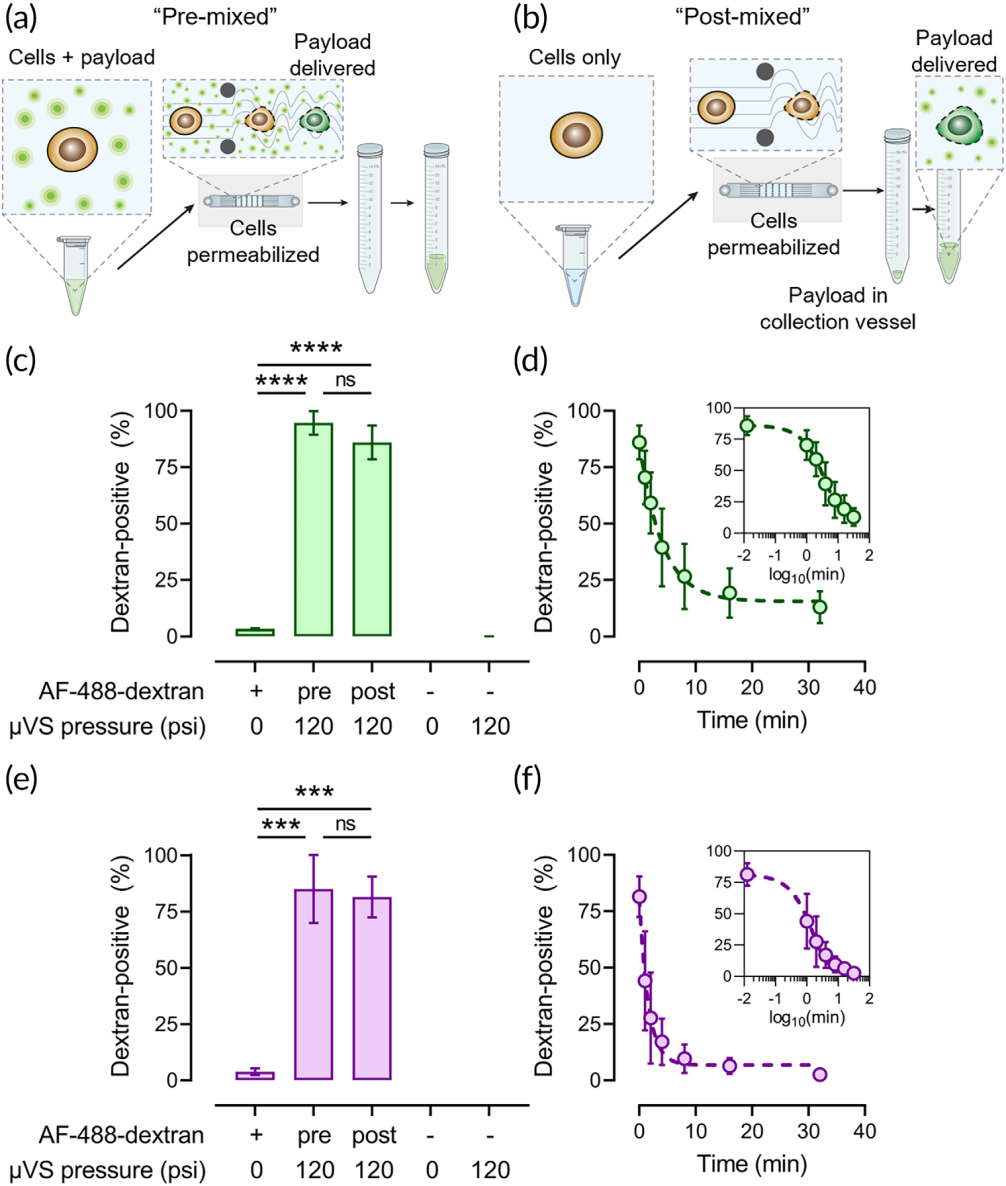
Induction of a short window of cell permeability after μVS‐processing. (a) Schematic representation of processing for cells that are pre‐mixed with fluorescently labeled dextran (average molecular weight = 3 kDa). (b) Schematic representation of processing for cells that are mixed with labeled dextran post‐μVS‐processing (“post‐mixed”). (c) Delivery efficiency of AF488‐dextran into HeLa cells in pre‐ and post‐mixed formats using μVS as determined by flow cytometry. ****P < 0.0001. (d) Membrane resealing kinetics of Hela cells after μVS‐processing. (e) Delivery efficiency of AF488‐dextran into HCT116 cells in pre‐ and post‐mixed formats using μVS. ***P < 0.001. (f) Membrane resealing kinetics of HCT116 cells after μVS‐processing.

To explore the possibility of using μVS for the ICD of cyclic peptides (~700–2000 Da), we used μVS to deliver AlexaFluor‐488‐labeled 3 kDa dextran into HeLa and HCT116 cells. We pre‐mixed cells with AF488‐dextran and processed them through μVS at 120 psi (Figure 1a), then monitored delivery by flow cytometry. Unsurprisingly, as μVS has previously been shown to deliver much larger macromolecular cargoes,^11^, ^12^ there was a substantial increase in the number of dextran‐positive cells (94.6 ± 5.2% and 85.0 ± 15.1%, in HeLa and HCT116, respectively) after μVS processing as compared to unprocessed cells treated with AF488‐dextran alone (3.3 ± 0.2% and 3.8 ± 1.5%, in HeLa and HCT116, respectively) (Figure 1c,e). We also sought to determine if cells maintain permeability after they have exited the μVS chip. Therefore, we tested μVS‐induced ICD by mixing AF488‐dextran with cells immediately post‐processing (Figure 1b). Since the flow rate through the device is ~8 ml/min, and the dead volume of the exit tubing is ~100 μl, the cells are expected to come into contact with the payload in ~0.75 s. There was only a very slight loss of delivery efficiency in both cell types using this approach (85.9 ± 7.5% and 81.5 ± 9.0%, in HeLa and HCT116, respectively) compared with the pre‐mixing methodology (Figure 1c, e). Importantly, there was minimal loss of cell viability in μVS‐processed cells (ranging from ~15–23% viability loss in HeLa, and ~1.7%–8.5% in HCT116) (Supplementary Figure 1). We next sought to determine the rate at which the cells reseal following collection. For these experiments, cells were processed similar to the post‐mix conditions described above and AF488‐dextran was then added at various timepoints post‐collection. Both HeLa and HCT116 cells rapidly resealed with a dramatic loss in delivery efficiency after several minutes (Figure 1d, f). When fitted to a one‐phase decay model, HeLa displayed a resealing *t*_1/2_ of 2.8 min (R^2^ = 0.9029) and HCT116 resealed with *t*_1/2_ of 1.1 min (R^2^ = 0.9020). Taken together, these data suggest that μVS can permeabilize cells to efficiently deliver low molecular weight macromolecules, and that cell membrane impermeability is re‐established within minutes.

### 3.2 Design of the DμVS system for high‐throughput microfluidic mechanoporation

**FIGURE 2:**
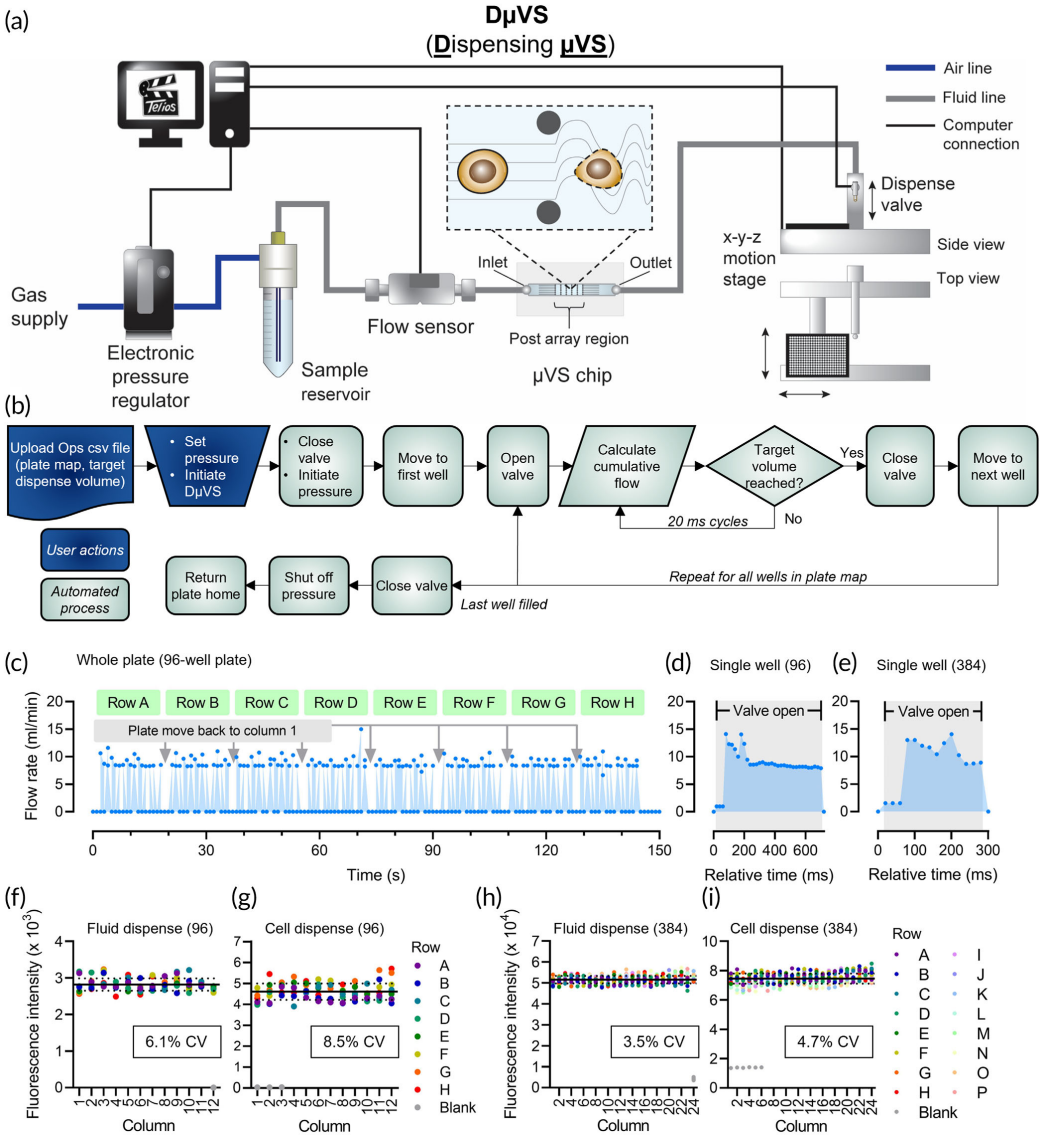
Overview of DμVS system and dispense characterization. (a) Component map of the DμVS system. An electronic pressure regulator controls the pressure that is applied to the cell suspension in the sample reservoir. When activated, the cell suspension flows through the flow sensor to the μVS chip where permeability is induced, then through the dispense valve, and finally to the designated well on a microplate. System components are controlled by a proprietary in‐house automation software package, Telios. (b) Software logic feedback loop (expanded details provided in the main text). (c) Example flow rate trace of dispensing into a 96‐well plate. An entire 96‐well plate can be filled in under 2.5 min when 100 μl is dispensed into each well. (d) Example flow rate trace (20 ms resolved) of DμVS dispensing into an individual well of a 96‐well plate. Flow rate is rapidly increased when the valve opens and stopped when the valve is closed. The valve is open for ~700 ms for 100 μl fill. (e) Flow rate trace (20 ms resolved) of DμVS filling into an individual well of a 384‐well plate. Valve is open for ~300 ms which dispenses 40 μl. (f) Plate‐wide fluorescence determination of FD20 solution (cell‐free) dispensed from DμVS into a 96‐well plate. (g) Plate‐wide fluorescence determination of CellTracker labeled HeLa cells DμVS‐processed into a 96‐well plate. (h) Plate‐wide fluorescence determination of FD20 solution (cell‐free) dispensed from DμVS into a 384‐well plate. (i) Plate‐wide fluorescence determination of CellTracker labeled HeLa cells DμVS‐processed into a 384‐well plate. In panels (f–i), plate‐wide average is indicated by the black horizontal line and dotted lines represent SD. The % CV values are indicated on each individual graph.

Given the potential utility of μVS to drug discovery, we sought to build a system capable of vector‐free ICD that is scaled to be compatible with microplate‐based screening methods. Since the delivery efficiency of μVS‐processed cells rapidly decreases after collection, we devised a strategy to quickly deliver permeabilized cells to multiple cargoes as quickly as feasible. Therefore, we converted the manual and labor‐intensive process of microfluidic sample handling to create a multiplexed (semi‐)automated process, by means of combining plate‐based dispensing with μVS (DμVS) (Figure 2a). We added four computer‐controlled components to the microfluidic method: (1) an electronic pressure regulator upstream of the sample reservoir, (2) a flow sensor downstream of the sample reservoir and just upstream of the μVS chip, (3) a microsolenoid valve‐based dispense tip on a z‐motor downstream of the chip, and (4) an x‐y motor stage that can accommodate and maneuver a standard microplate beneath the dispense tip (Figure 2a). All four components are synced up and controlled through our proprietary automation software, Telios.^14^ The electronic pressure regulator allows the user to choose and initiate the applied pressure at the start of an experimental run. The inline flow sensor provides rapid flow feedback to the system, thereby controlling for two phenomena that can occur when cell suspensions pass through microfluidic chips at this size scale and flow regime: (1) very fast clogging/unclogging events at high frequency and (2) slow clogging from accumulation of cells/debris within the chip. The dispense valve allows for precise stopping of the flow and the x‐y stage moves the microplate to position beneath the dispense tip. The basic logic process operated by the Telios system is shown in the flowchart in Figure 2b. The user uploads an operations file, which indicates the target volume for each well, and indicates the desired pressure for cell processing. Once the user initiates DμVS, the rest of the process is automated via Telios. The dispense valve is put into the closed state and the desired pressure is applied. The x‐y stage positions the microplate so that the first well is beneath the dispense tip and the valve is opened. As the cell suspension flows through the system, the flow rate data is fed back to the software via the flow sensor in 20 ms loops. After the target volume is achieved, the dispense valve is closed, the x‐y stage positions the next well under the dispense tip, and the valve is opened to repeat the process. The flow rate data are logged throughout the process (Figure 2c–e). To characterize the accuracy of this semi‐automated process, we processed a cell‐free solution of 20 kDa FITC‐dextran (FD20) into both 96‐ and 384‐well plates and then measured the percent coefficients of variation (% CV) in fluorescent signal (Figure 2f, h; 96‐well = 6.1% CV, 384‐well = 3.5% CV). The fluid was processed with an applied pressure of 120 psi to mimic the conditions where cell permeability is achieved (as shown above and in previous reports^11^, ^12^). Likewise, we characterized the well‐to‐well consistency of processing and dispensing a cell suspension at 120 psi. In this case fluorescently labeled HeLa cells were DμVS‐processed to 96‐ and 384‐well plates, and the resulting fluorescence was recorded across the plate (Figure 2g, i; 96‐well = 8.5% CV, 384‐well = 4.7% CV). These results indicate that our DμVS configuration, with the added components to semi‐automate and increase throughput of μVS, can achieve good well‐to‐well dispensing homogeneity of solutions and cell suspensions that undergo hydrodynamic conditions that are conducive to mechanoporation.

### 3.3 Intracellular delivery of cyclic peptides into DμVS‐processed cells

**FIGURE 3:**
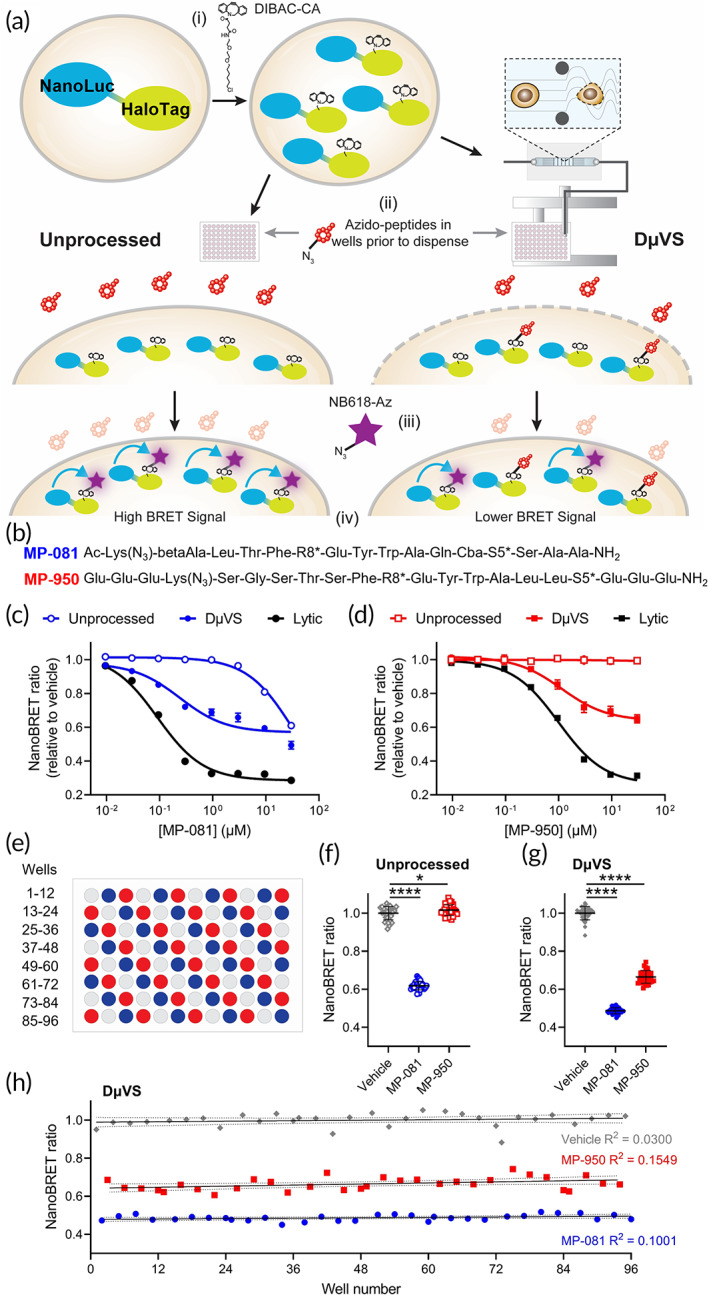
DμVS‐enhanced delivery of cyclic peptides characterized with NanoClick assay. (a) Schematic representation of how the NanoClick assay was used to quantify relative peptide delivery enhancement of DμVS. NanoLuc‐HaloTag expressing HeLa cells were treated with DIBAC‐CA (i), after which the cells were transferred to 96‐well plates containing cyclic azido‐peptides either by standard pipetting or DμVS dispensing (ii). After incubation, cells were treated with NB618‐Az (iii) and then BRET signal was measured upon addition of NanoLuc substrate (iv). Increased azido‐peptide delivery leads to a decreased BRET signal. (b) Sequences of azido‐peptides used to test DμVS‐based delivery. S5: (S)‐2‐(4′‐pentenyl) alanine; R8: (R)‐2‐(7'octenyl) alanine; “*” site of stapling. MP‐081 exhibits moderate permeability whereas MP‐950 is cell impermeable, primarily due to multiple glutamic acids at both its C‐ and N‐terminus. (c, d) Dose response curves of MP‐081 (c) and MP‐950 (d) in the NanoClick assay without cell permeabilization (unprocessed), with DμVS processing, and with digitonin treatment (lytic). (e) Plate map design for the data shown in panels (f–h). Gray wells represent vehicle, blue wells represent 30 μM MP‐081 and red wells represent 30 μM MP‐950. Aggregate NanoBRET data of cells without permeabilization (unprocessed) (f) and with DμVS processing (g). The CV values for DμVS processed cells were: 3.52% for vehicle treatment, 3.24% for MP‐081 and 5.01% for MP‐950. *P < 0.05, ****P < 0.0001. (h) Data from panel (g) plotted by well position on plate. Data were fitted with a linear regression model and 95% confidence interval is shown (solid and dotted lines, respectively).

In order to characterize DμVS‐based ICD of peptides in a high‐throughput amenable cell‐based permeability assay, we employed the NanoClick technology.^16^ NanoClick is a target‐agnostic permeability assay that utilizes in‐cell copper‐free click chemistry,^18^ HaloTag labeling,^19^ and NanoBRET technology^20^ (Figure 3a). We selected for analysis two azido‐analogs of ATSP‐7041,^21^ a peptide that targets the PPI between two cytoplasmic proteins, p53 and MDM2: (1) MP‐081, a cell‐permeable positive control peptide with MDM2 binding *K*_D_ of 19 nM (biochemical) and EC_50_ of 0.42 μM in the p53 reporter assay (cellular),^17^ and (2) MP‐950, a cell‐impermeable peptide that shows high‐affinity binding to MDM2 (*K*_D_ = 2.3 nM) but does not display cellular activity in the p53 reporter assay (EC_50_ > 50 μM; Figure 3b).^16^

HeLa cells expressing a NanoLuc‐HaloTag construct were treated with DIBAC‐CA so that the HaloTag domain reacts with the chloroalkane linker, rendering close proximity of the DIBAC group and NanoLuc domain (Figure 3a, [i]). These cells were then processed by DμVS directly into plates containing pre‐plated peptides (Figure 3a, [ii]). In parallel, unprocessed cells (non‐permeabilized control) and cells treated with digitonin (lytic control) were exposed to the same peptide conditions as the DμVS‐processed cells. When azido‐peptides enter the cell, they undergo a strain‐promoted azide‐alkyne click reaction with the DIBAC group on the NanoLuc‐HaloTag construct. Cells were then treated with the azido‐modified NanoBRET fluorophore (NB618‐Az), which reacts with any remaining unoccupied DIBAC groups (Figure 3a, [iii]). Thus, a BRET signal is generated in inverse proportion to the amount of cytosolic peptide (Figure 3a, [iv]). When cells were unprocessed, MP‐081 showed moderate permeability while MP‐950 showed no permeability, similarly to previous observations^16^ (Figure 3c, d). However, when cells were processed through the DμVS system, a dose‐dependent reduction in BRET signal is observed with both azido‐peptides, demonstrating ICD and “click” reactivity with the DIBAC groups (MP‐081 EC_50_ = 0.24 μM, MP‐950 EC_50_ = 1.14 μM; Figure 3c, d). As a positive control we used a lytic format of the assay where cells were treated with digitonin, a detergent that permeabilizes the cell membrane. With this treatment, we also observed dose‐dependent BRET signal inhibition for both peptides (MP‐081 EC_50_ = 0.09 μM, MP‐950 EC_50_ = 0.99 μM; Figure 3c, d). While the EC_50_ values for both peptides were similar between DμVS and the lytic assay formats, the lytic treatment contributed a greater max inhibition of BRET signal (~73% lytic vs. ~40% DμVS). This is likely the result of the timescale of permeability, where DμVS induces transient porosity that rapidly reseals within several minutes whereas digitonin induces sustained permeability throughout the entire assay incubation.

After observing the DμVS‐induced enhanced delivery of azido‐peptides in a dose‐dependent fashion, we utilized the NanoClick assay to characterize azido‐peptide delivery efficiency as a function of position on the plate—we sought to understand any variance that the DμVS process might introduce in this cell‐based assay and determine if there are any locational effects. We plated high concentrations of both MP‐081, MP‐950, as well as DMSO (vehicle) as shown (Figure 3e). The permeabilized cells were dispensed to the full 96‐well plate within 130 sec (data not shown). In the unprocessed control we observed % CV of 3.53% for vehicle treatment, 3.43% for MP‐081 and 2.79% for MP‐950 (Figure 3f). For DμVS, the observed % CVs were 3.52% for vehicle treatment, 3.24% for MP‐081 and 5.01% for MP‐950 (Figure 3g). This suggests that DμVS processing has very little effect on the intrinsic variability of the assay (unchanged variation in the vehicle and MP‐081 treatments). In addition, the location on the plate has little impact on the variance of cytosolic delivery of MP‐950 via DμVS (well number vs. NanoBRET R^2^ = 0.1549; Figure 3h). These data suggest that DμVS can be used for robust, repeatable ICD of cyclic peptides.

### 3.4 MP‐950 exhibits cellular activity after DμVS processing in a p53 reporter assay

**FIGURE 4:**
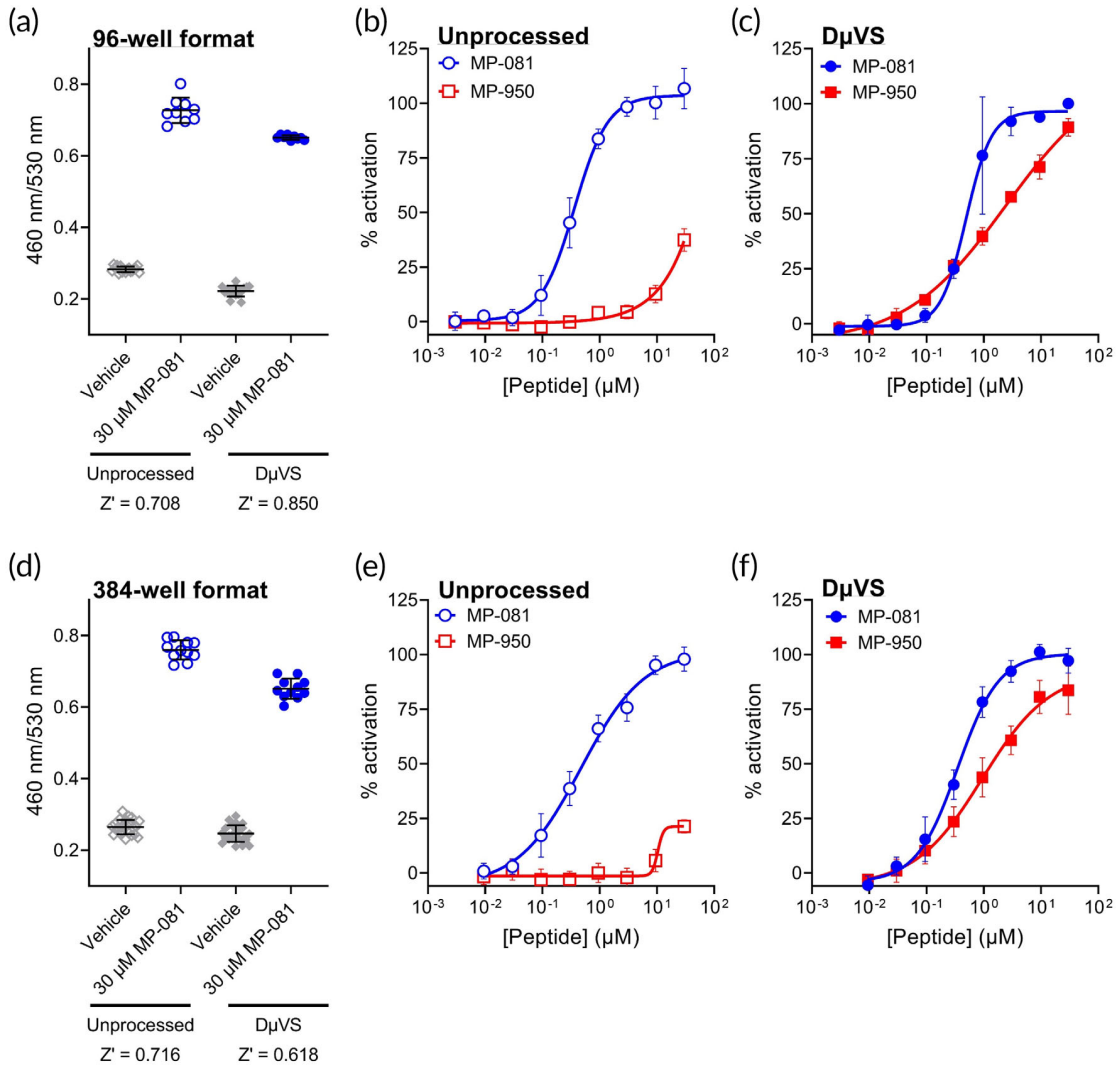
DμVS enables an impermeable cyclic peptide to drive a functional response in a p53 reporter assay. (a) Window of the 96‐well format p53 reporter assay in unprocessed and DμVS conditions. The ratiometric readout normalizes p53‐controlled β‐lactamase activity (460 nm) to total cell number (530 nm). *Z*‐prime values for both assay formats are shown beneath the x‐axis. (b) Dose response curves of MP‐081 and MP‐950 in the unprocessed 96‐well assay format. (c) Dose response curves of MP‐081 and MP‐950 in the DμVS 96‐well assay format. (d) Window of the 384‐well format p53 reporter assay in unprocessed and DμVS conditions. *Z*‐prime values for both assay formats are shown beneath the x‐axis. (e) Dose response curves of MP‐081 and MP‐950 in the unprocessed 384‐well assay format. (f) Dose response curves of MP‐081 and MP‐950 in the DμVS 384‐well assay format.

Once we demonstrated that ICD of the impermeable cyclic peptide MP‐950 could be enhanced with the DμVS method, we next sought to determine if this delivery is sufficient to drive a functional effect toward disrupting the p53/MDM2 PPI. For these experiments, we leveraged an HCT116‐based cell line containing a β‐lactamase reporter gene that is responsive to p53 activity. This assay has previously been used to characterize the cellular activity of p53/MDM2 peptides, including MP‐081 and MP‐950, where the former displayed good cell potency (EC_50_ = 0.42 μM) and the latter showed no activity.^16^, ^17^ The assay, which is typically performed on adherent cells, was adapted to accommodate the DμVS process where cells must be in suspension to flow through the device, as outlined in “Methods”. Similar to the NanoClick assay above, cells were DμVS‐processed directly onto pre‐plated peptides. After incubation, cells are treated with a FRET‐based fluorescent β‐lactamase substrate to generate a ratiometric readout of p53‐regulated gene expression per cell number. In 96‐well format, the assay performs well with the above modifications, as both the unprocessed cells (cells in suspension but not flowed through DμVS) and the DμVS‐processed conditions displayed good Z‐prime scores when comparing vehicle treatment (negative control) with a high concentration of MP‐081 (positive control; Figure 4a). In the unprocessed conditions (i.e. no DμVS), MP‐081 and MP‐950 had similar potencies as described in previous reports^16^, ^17^ (Figure 4b; MP‐081 EC_50_ = 0.36 μM, MP‐950 EC_50_ > 30 μM). Following DμVS processing however, MP‐950 showed a clear dose response and low‐micromolar potency (EC_50_ = 2.4 μM; Figure 4c), while the potency of the permeable MP‐081 was largely unchanged (EC_50_ = 0.49 μM). These data indicate that this high‐affinity MDM2‐binder is capable of properly engaging the target and driving efficacy in the native cell environment when its cellular permeability is increased.

After showing that the DμVS system yields robust results in cell‐based assays in 96‐well format, we were interested to see if we could further increase the throughput of DμVS by performing the p53‐reporter assay in a 384‐well format. In this higher throughput format, six replicates of dose titrations for each peptide could be tested (Supplementary Figure 2). Similar to the 96‐well format, the assay showed good signal‐to‐noise and Z‐prime values with the necessary accommodations to implement DμVS (Figure 4d). The unprocessed conditions also yielded expected results (Figure 4e; MP‐081 EC_50_ = 0.47 μM, MP‐950 EC_50_ > 30 μM). However, similar to our observations in the 96‐well format, MP‐950 showed a large increase in activity in a dose‐dependent manner when cells undergo DμVS processing (EC_50_ = 0.95 μM; Figure 4f), whereas MP‐081 activity was unaffected (EC_50_ = 0.35 μM; Figure 4f).

### 3.5 Variable activity of p53/MDM2 cyclic and linear peptides in a cellular context

**FIGURE 5:**
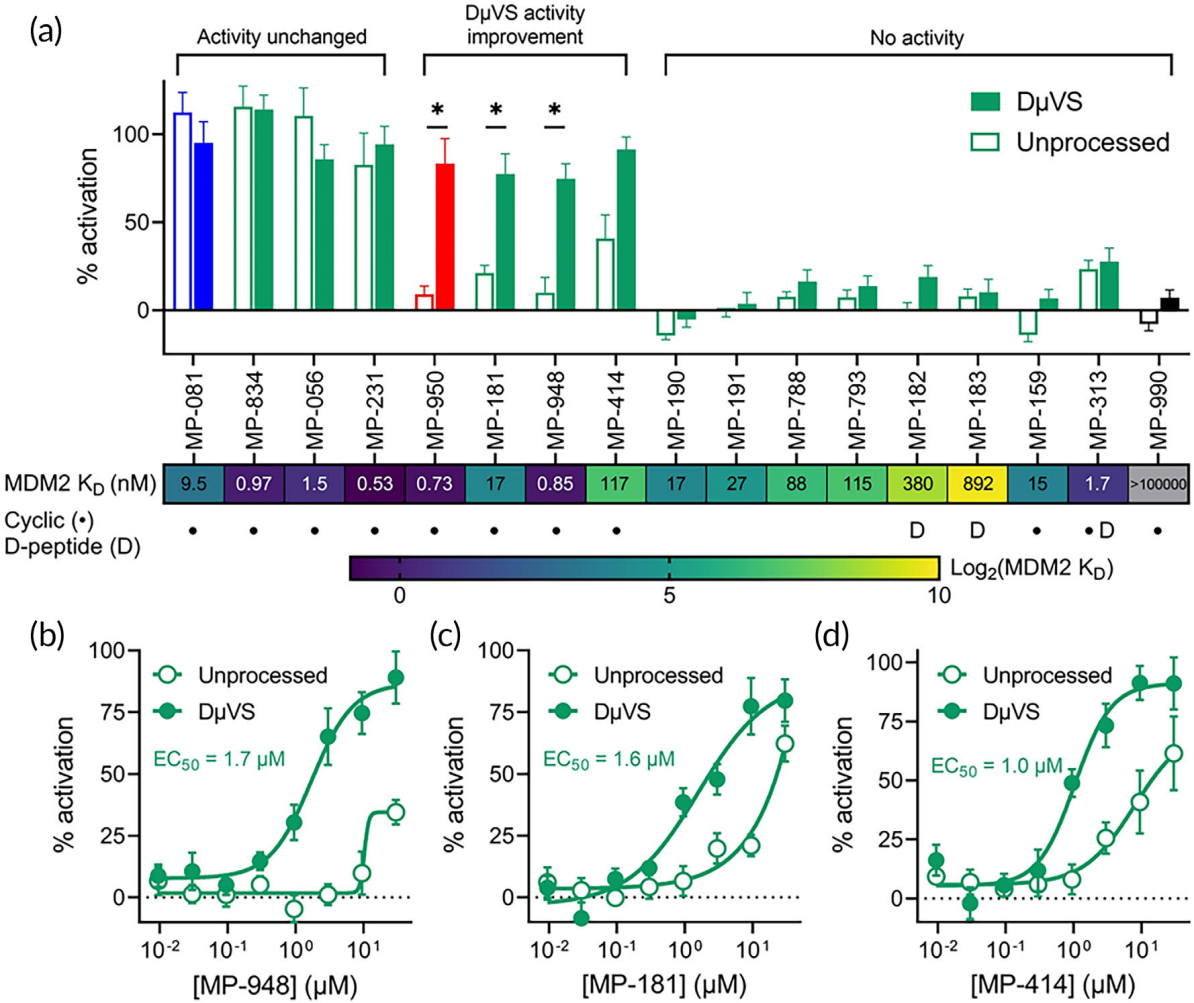
DμVS‐p53 reporter screening of additional p53/MDM2 peptides. (a) Activity of 17 peptides at 10 μM in the p53 reporter assay with and without DμVS. Blue bars represent the positive control peptide (MP‐081), red bars represent the intracellular delivery control peptide (MP‐950), and black bars represent the non‐binding negative control peptide (MP‐990). *P < 0.001. Peptides were reordered on graph into three classes: (i) activity unchanged between methods, (ii) DμVS‐improved activity, and (iii) no activity in either method. The heat map beneath the graph labels indicates the MDM2 binding affinity of each peptide. (b–d) Selected dose response curves of validated hits in the DμVS‐p53 reporter assay: MP‐948 (b), MP‐181 (c), and MP‐414 (d). The EC_50_ value displayed on the graph is from the DμVS‐based assay format.

We next sought to determine the generalizability of the DμVS technique across different p53/MDM2 peptides that have a broad range of activities in both the non‐permeabilized p53 reporter assay and a cell‐free MDM2‐binding assay. We assessed an additional 15 peptides from previously reported literature,^17^, ^21^, ^22^, ^23^, ^24^, ^25^ including a non‐binding cyclic peptide as a negative control (MP‐990; Supplementary Table 1). We measured p53 activation at 10 μM of each peptide and observed that the peptides generally fall into three groups: (i) peptides whose activities are unchanged by DμVS processing, (ii) peptides that show enhanced activity with DμVS‐processed cells, and (iii) peptides that show no activity in either format (Figure 5a). The control peptides behaved as expected—MP‐081 displayed no difference in activity, MP‐950 showed improved activity following DμVS, and the non‐binding peptide MP‐990 showed no activity in either format (Figure 5a). In addition to MP‐081, three other peptides had activity that was unaffected by DμVS. Of the remaining 13 peptides, only three had enhanced activity in the p53 reporter assay when the cells were processed in the DμVS system (in addition to MP‐950; Figure 5a). These activities were confirmed through dose–response experiments where low micromolar potency was observed (Figure 5b–d). Nine peptides showed no cell‐based activity even in the permeabilized cell format, despite several of them binding to MDM2 with *K*_D_s in the nanomolar range (Figure 5a). These data suggest that binding to MDM2 is not sufficient for p53 activation. This underscores how the DμVS technique can improve understanding of complex modalities, such as cyclic peptides, and intracellular target engagement.

## 4 DISCUSSION

Combinatorial cyclic peptide libraries are particularly well‐suited for discovering potent binders of difficult intracellular PPIs.^26^ However, conducting SAR studies with these molecules is fraught with challenges as these molecules typically do not have sufficient cell permeability and do not follow normal druglikeness conventions (e.g., Lipinski's rule of five^27^). Therefore, vector‐free ICD approaches with suitable throughput for cell‐based assay screening may be beneficial in building knowledge around functional target engagement of impermeable cyclic peptides in a native cellular environment. To address this need, we built the DμVS platform, which utilizes μVS‐based mechanoporation integrated with microplate‐based dispensing to perform transiently permeabilized cell‐based assays with cyclic peptides in a high‐throughput format.

Our first step toward increasing the throughput/multiplexity of microfluidic mechanoporation was to understand the timeframe of which cells are permeabilized. We performed experiments where cells were processed through a μVS chip, and then exposed to a fluorescent payload at pre‐determined timepoints. Importantly, the cell viability loss due to μVS was modest and appeared to be at a similar level to previous reports with μVS,^11^, ^12^ as well as another microfluidic cell shearing method,^28^ and cell constriction.^29^, ^30^, ^31^, ^32^ We characterized the decay in delivery efficiency over time post‐μVS, and showed it to be on a similar timescale as the resealing of plasma membranes observed after cell squeezing.^33^ This defined a brief time window for us to deliver permeabilized cells to respective payloads after which there is an exponential decrease in delivery efficiency over time. Because μVS is performed at a high flow rate (~8 ml/min) and the dead volume of tubing downstream of the chip is relatively low (~100–200 μl), we surmised that permeabilized cells can be delivered to a multiplex of payloads with minimal loss in delivery efficiency, if the right fluidic handling is built around it.

The DμVS system uses a μVS device, which has previously been shown to deliver large macromolecules into primary human T cells,^11^, ^12^ and incorporates additional parts, including an electronic pressure regulator, a flow sensor, a dispense valve, and two motion‐control gantries. These components rely on custom automation scheduling software (Telios) to enable synchronized process steps and decision making which are critical to DμVS. With these capabilities in hand, we were able to deliver a novel system that integrates a microfluidic mechanoporation chip with microplate dispensing that can be leveraged for compound screening in cell‐based assays. To ensure that the system had suitable dispensing accuracy for these applications, we characterized the dispensing of both cell‐free fluids and cell suspensions, and demonstrated that the DμVS system yields good homogeneity of dispensing across 96‐ and 384‐well plates.

To pressure‐test the ability of the DμVS system to permeabilize cells to deliver cyclic peptides, we used a recently developed click chemistry‐based permeability assay called NanoClick.^16^ Using this assay, we demonstrated that DμVS could enhance the ICD of two model p53/MDM2 peptides (MP‐081 and MP‐950) in a concentration‐dependent manner. The ICD of MP‐950 was especially encouraging, as this peptide is highly‐negatively charged (−6 net charge), due to having three glutamic acid residues at both the C‐ and N‐termini. We also used the NanoClick assay to demonstrate that there are no microplate locational effects on peptide delivery efficiency following DμVS.

After demonstrating that DμVS facilitates the ICD of these molecules, we confirmed that they retained their functional activity after DμVS using a p53 reporter assay. While MP‐950 has no cell‐based activity due to its low permeability, DμVS processing significantly increased its activity resulting in low micromolar EC_50_. The potency of MP‐081, the positive control peptide in this assay, was unchanged, suggesting that the cellular activity of MP‐081 is not limited by permeability, consistent with our data and previous data^16^ showing that this peptide has intrinsic cellular permeability. Importantly, these results also show that the p53 pathway, and its interaction with MDM2, remain intact after DμVS processing. Additionally, we showed similar assay robustness in 96‐ and 384‐well formats, enabling us to characterize target engagement of these cyclic peptides at an even higher throughput. Microfluidic cell squeezing has previously been reported for target engagement studies with poorly permeable small molecules (Janus kinase inhibitors), although those studies suffered from low throughput and analytical capability.^34^ In the current work, we show that the DμVS system achieves this application at an unprecedented scale and throughput in two orthogonal cell‐based assays.

We used our high‐throughput DμVS‐p53 reporter (target engagement) assay to characterize an expanded set of MDM2‐binding peptides with a range of biochemical binding and cell‐based activities. These peptides fell into three groups: (i) peptides whose activities are unchanged by DμVS processing (i.e., functional permeable), (ii) peptides that show enhanced activity with DμVS‐processed cells (i.e., functional impermeable), and (iii) peptides that show no activity in either format despite having potent MDM2 binding affinity (i.e., non‐functional). Our results suggest that peptides in the “functional impermeable” group are good candidates for chemical optimization of cell permeability. Conversely, peptides in the “non‐functional” group could inform SAR efforts, as compounds with structures that are not associated with functional activity. One observation that stood out is that the “non‐functional” group was dominated by linear peptides (6/8, Figure 5a, Supplementary Table 1). It is well known cyclization of peptides confers greater metabolic stability in comparison to their linear counterparts.^35^ While we did not assess peptide stability in this study, this could be one explanation for the lack of activity seen with these peptides. This, however, would not account for the lack of activity observed from MP‐182 and MP‐183, as these linear D‐peptides should be resistant to protease degradation, although they also had weaker MDM2 affinity. The majority of the “functional impermeable” peptides also have higher binding affinities, in agreement with the prevailing opinion that a high affinity (*K*_D_ < 10 nM)^36^ is associated with a higher potency. However, this was not always the case: MP‐414 showed functional activity (DμVS‐p53 reporter EC_50_ = 1.0 μM, MDM2 binding *K*_D_ = 117 nM) and MP‐313 was a very high‐affinity peptide that showed no p53 reporter activity improvement (MDM2 binding *K*_D_ = 1.7 nM). The high throughput of the DμVS platform may allow us to address such discrepancies in the future.

The development of the DμVS system opens up several additional areas of investigation beyond the scope of our current effort. First, it may allow us to better understand the physicochemical properties, beyond molecular size/weight, that dictate cell permeability and the ability of a cyclic peptide to diffuse into a permeabilized cell. In addition, it is unclear what intracellular payload concentration can be achieved with DμVS, and how it might be impacted by various factors such as extracellular concentration, cell type, and nature of the payload. Relating to the latter, it would be important to understand the upper limits of size (and corresponding delivery efficiency) of molecular payloads delivered in the “post‐mixing” format that DμVS leverages. While μVS has been shown to deliver large molecular payloads of ~160 kDa (Cas9‐RNP) and ~300 kDa (EGFP‐encoding mRNA), these were in a pre‐mixed format, and their delivery efficiency in a post‐mix format is uncharacterized as of yet.^11^, ^12^ Because of the post‐mixing format of DμVS, and since larger plasma membrane pores (>100 nm) have been demonstrated to repair more rapidly,^2^, ^37^, ^38^ there could be reduced delivery efficiency using DμVS as payload molecular weight increases—although the nature of pores (e.g., size, number) formed by μVS is incompletely characterized to‐date. Finally, it is likely that further tuning of parameters such as operating pressure (and therefore flow rate), temperature during or post‐DμVS processing, and cell suspension buffer composition could maximize delivery efficiency and with minimal impact on cell function/viability.

A limitation of our study was that DμVS was not compared head‐to‐head with another physical membrane‐disruption‐mediated ICD method, such as cell squeezing,^32^ other cell shearing methods,^28^ or conventional electroporation. While there are microplate‐based electroporation apparatuses commercially available, to the best of our knowledge none of these approaches have been demonstrated for cyclic peptide delivery and downstream cell‐based screening assays, so the utility of these other techniques in this application is currently unclear. It is our hope that additional methods toward this end will be uncovered and developed.

## 5 CONCLUSION

There is currently a significant effort toward improving the drug‐like properties of macrocyclic peptides, including cellular permeability.^35^, ^39^ In addition to modifying the peptides themselves, advances in targeted delivery via nanocarriers,^40^, ^41^, ^42^, ^43^, ^44^, ^45^ cell‐penetrating peptides,^7^ antibody‐drug conjugates,^46^ and more, may enable formulation‐based strategies to deliver peptides intracellularly to engage with targets of interest that may be difficult to drug with small molecules. The DμVS approach we describe herein increases the probability of success of these synthetic‐ and formulation‐based approaches by yielding early demonstration of peptide functional activity. DμVS also complements orthogonal efforts in the field to bias mRNA display codons and incorporate non‐proteinogenic amino acids for early selection of peptides with higher likelihood of permeability.^47^, ^48^ Furthermore, while we focused on cyclic peptides in this study, the DμVS system may be useful with various emerging drug modalities (such as PROTACs, DUBTACS, molecular glues, etc.),^49^, ^50^ as well as with more traditional small molecule programs which are not always exempt from permeability issues. In closing, we used a multidisciplinary approach integrating a microfluidic‐based process with high‐throughput technology to build a system that will add to the toolkit of technologies aimed at enabling drug discovery for notoriously difficult intracellular targets.

## AUTHOR CONTRIBUTIONS

**Stephen H. Kasper:** Conceptualization (lead); formal analysis (equal); investigation (equal); methodology (equal); resources (equal); software (supporting); supervision (lead); visualization (lead); writing – original draft (lead); writing – review and editing (equal). **Stephanie Otten:** Investigation (equal); methodology (equal); resources (equal); software (supporting); visualization (supporting); writing – original draft (supporting); writing – review and editing (equal). **Brian Squadroni:** Conceptualization (lead); formal analysis (equal); methodology (equal); resources (equal); software (equal); writing – review and editing (equal). **Cionna Orr‐Terry:** Investigation (equal); methodology (equal); writing – review and editing (supporting). **Yi Kuang:** Investigation (equal); methodology (equal); writing – review and editing (supporting). **Lily Mussallem:** Methodology (equal); resources (equal); software (equal); writing – review and editing (supporting). **Lan Ge:** Methodology (equal); resources (equal); writing – review and editing (supporting). **Lin Yan:** Resources (equal); writing – review and editing (supporting). **Srinivasaraghavan Kannan:** Resources (equal); writing – review and editing (supporting). **Chandra S. Verma:** Resources (equal); writing – review and editing (supporting). **Christopher J. Brown:** Resources (equal); writing – review and editing (supporting). **Charles W. Johannes:** Resources (equal); writing – review and editing (equal). **David P. Lane:** Resources (equal); writing – review and editing (supporting). **Arun Chandramohan:** Formal analysis (equal); investigation (equal); methodology (equal); resources (equal); writing – review and editing (supporting). **Anthony W. Partridge:** Formal analysis (equal); investigation (equal); methodology (equal); resources (equal); writing – review and editing (equal). **Lee R. Roberts:** Conceptualization (equal); supervision (equal); writing – review and editing (supporting). **Hubert Josien:** Data curation (equal); resources (equal); writing – review and editing (supporting). **Alex G. Therien:** Resources (equal); supervision (supporting); writing – review and editing (equal). **Erik C. Hett:** Conceptualization (equal); resources (equal); supervision (equal); writing – review and editing (supporting). **Bonnie J. Howell:** Resources (equal); supervision (equal); writing – review and editing (equal). **Andrea Peier:** Methodology (equal); resources (equal); supervision (supporting); writing – review and editing (equal). **Xi Ai:** Data curation (equal); methodology (equal); resources (equal); supervision (supporting); writing – original draft (supporting); writing – review and editing (equal). **Jason Cassaday:** Conceptualization (lead); formal analysis (equal); investigation (equal); methodology (equal); resources (equal); software (equal); supervision (lead); visualization (supporting); writing – original draft (equal); writing – review and editing (equal).

## CONFLICT OF INTEREST STATEMENT

All authors that are present or former employees of Merck Sharp & Dohme LLC, a subsidiary of Merck & Co., Inc., Rahway, NJ or MSD International, Singapore may hold stocks and/or stock options in Merck & Co., Inc., Rahway, NJ. Srinivasaraghavan Kannan and Chandra S. Verma are co‐founders of Sinopsee Therapeutics and Aplomex; the current work does not have a conflict.

### PEER REVIEW

The peer review history for this article is available at https://www.webofscience.com/api/gateway/wos/peer-review/10.1002/btm2.10542.

## Supporting information


**Data S1.** Supporting Information.

## Data Availability

The data that support the findings of this study are available from the corresponding author upon reasonable request.
