# Supplementary material for: A high‐throughput microfluidic mechanoporation platform to enable intracellular delivery of cyclic peptides in cell‐based assays
Source: Bioeng Transl Med. 2023 May 13;8(5):e10542. doi: 10.1002/btm2.10542 (PMC10487316; doi:10.1002/btm2.10542)
Supplement: Supplementary file 1 — Data S1. Supporting Information. [file BTM2-8-e10542-s001.docx]

***Supporting Information***


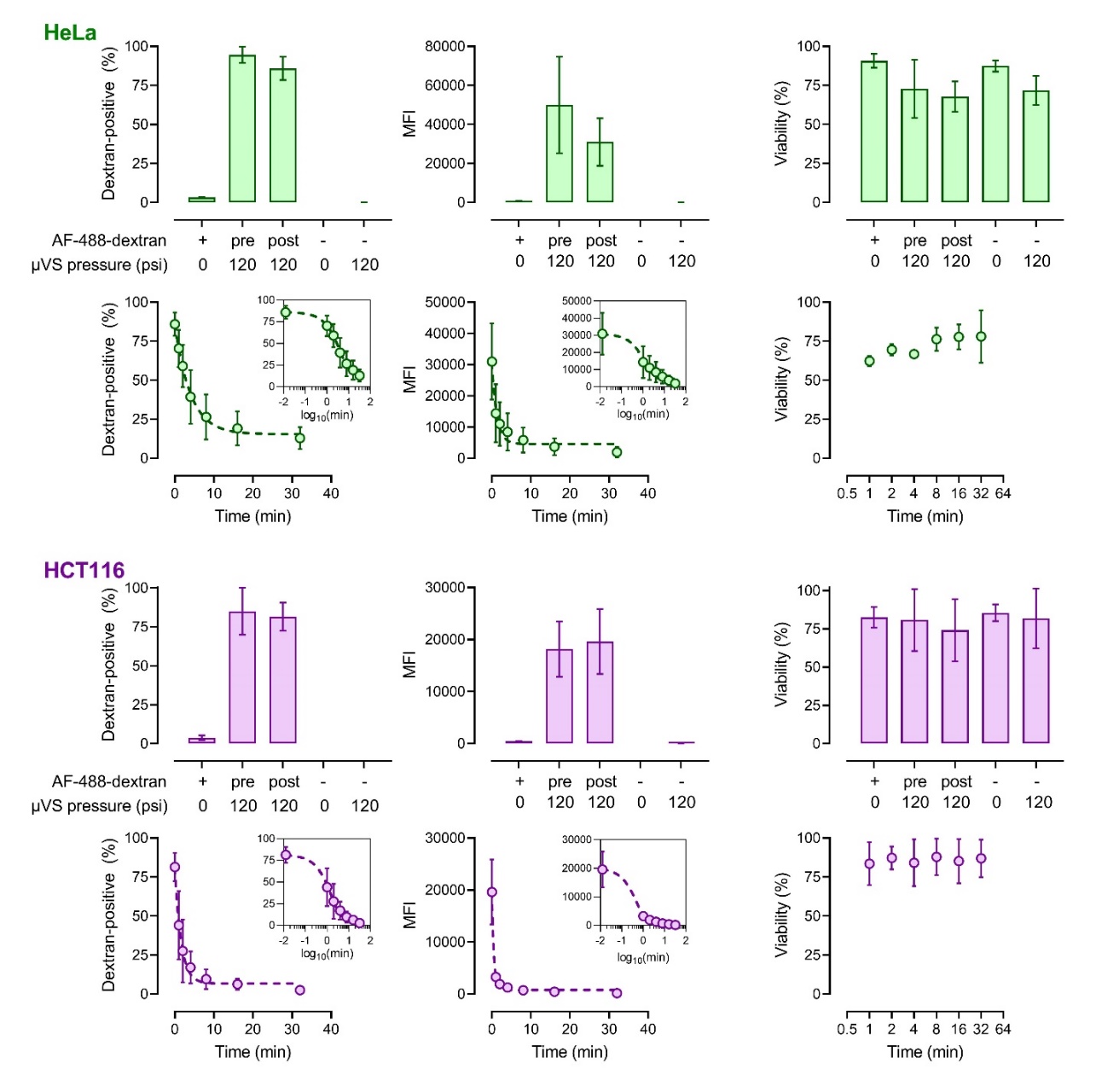


**Supplementary Figure 1.** Expanded data on effects of µVS-processing on cell permeability to 3 kDa AF-488 and viability. Data from HeLa cells are in green (top 6 panels) and data from HCT116 cells are in purple (lower 6 panels). Mean fluorescence intensity (MFI) and viability are shown in the middle and right column, respectively. The first and third row display the data from the pre-mixed versus post-mixed comparison. The second and fourth row display data from the time course expierments.


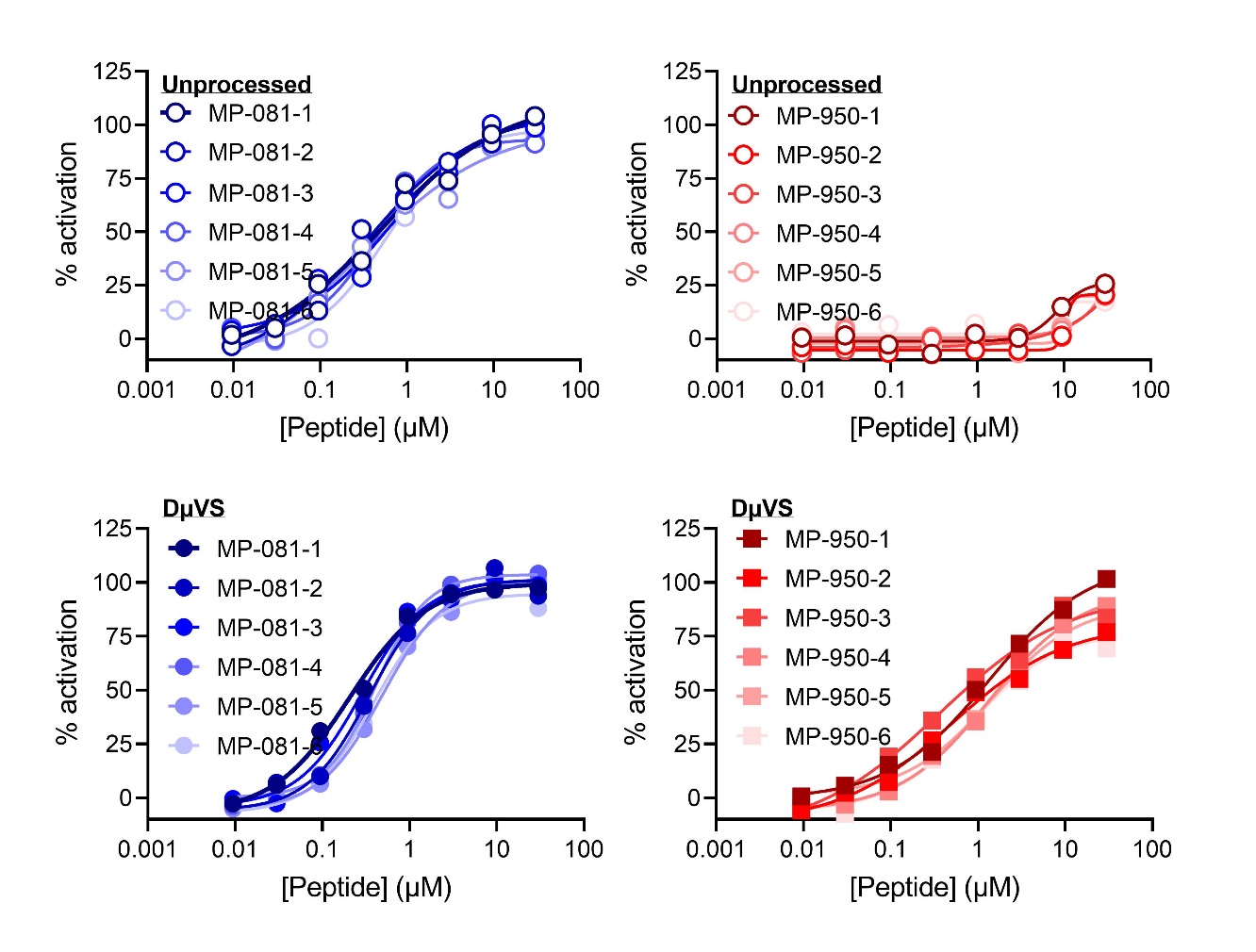


**Supplementary Figure 2.** Six replicate dose titrations from a single experiment. Unprocessed cells (no DµVS) are shown on the top row. DµVS-processed cells are shown in the bottom row. The permeable peptide, MP-081, is shown in the left column, whereas the impermeable peptide, MP-950, is shown in the right column.

**Supplementary Table 1.** Sequences of p53/MDM2 peptides used in this study.

| **Peptide** | **Sequence** |
| --- | --- |
| MP-081 | Ac-Lys(N_3_)-betaAla-Leu-Thr-Phe-R8*-Glu-Tyr-Trp-Ala-Gln-Cba-S5*-Ser-Ala-Ala-NH_2_ |
| MP-834 | Ac-Lys(N_3_)-betaAla-Leu-Thr-Phe-R8*-Glu-Tyr-Trp-Ala-Gln-Cba-S5*-Glu-Ala-Ala-Ala-Ala-DAla-NH_2_ |
| MP-056 | Ac-Lys(N_3_)-betaAla-Leu-Thr-Phe-R8*-Glu-Tyr-Trp-Ala-Gln-Cba-S5*-Ala-DAla-Ala-DAla-Ala-DAla-NH_2_ |
| MP-231 | H-R5*-DAla-DTrp(6-F)-DTyr-B5**-DAsn-DPhe(4-CF3)-DGlu-DLys-DLeu-DLeu-R8*-DAla-DAla-DAla-DAla-DAla-DAla-NH_2_ |
| MP-950 | NH2-Glu-Glu-Glu-Lys(N_3_)-Ser-Gly-Ser-Thr-Ser-Phe-E8*-Glu-Tyr-Trp-Ala-Leu-Leu-S5*-Glu-Glu-Glu-NH_2_ |
| MP-948 | NH2-Glu-Glu-Glu-Lys(N_3_)-Ser-Gly-Ser-Thr-Ser-Phe-R8*-Glu-Tyr-Trp-Ala-Leu-Leu-S5*-NH_2_ |
| MP-181 | H-Glu-Glu-Lys(N_3_)-Ser-Gly-Ser-Thr-Ser-Phe-R8*-Glu-Tyr-Trp-Ala-Leu-Leu-S5*-NH_2_ |
| MP-414 | H-Lys(N_3_)-betaAla-Leu-Thr-Phe-R8*-Glu-Tyr-Trp-Ala-Gln-Cba-S5*-Ser-Ala-Ala-Lys(mPEG24)-NH_2_ |
| MP-190 | Ac-Leu-Thr-Phe-Glu-Glu-Tyr-Trp-Ala-Gln-Leu-Thr-Ser-NH_2_ |
| MP-191 | Ac-DLeu-Thr-Phe-Glu-Glu-Tyr-Trp-Ala-Gln-Leu-Thr-Ser-NH_2_ |
| MP-788 | Ac-Leu-Thr-Phe-Aib-Glu-Tyr-Trp-Gln-Leu-Cba-Aib-Ser-Ala-Ala-OH |
| MP-793 | Ac-Leu-Thr-Phe-Glu-Glu-Tyr-Trp-Ala-DGln-Leu-Thr-Ser-NH_2_ |
| MP-182 | H-DThr-DAla-DTrp-DTyr-DAla-DAsn-DPhe-DGlu-DLys-DLeu-DLeu-DArg-NH_2_ |
| MP-183 | Ac-DThr-DAla-DTrp-DTyr-DAla-DAsn-DPhe-DGlu-DLys-DLeu-DLeu-DArg-NH_2_ |
| MP-159 | Ac-Thr-Ser-(ɑ-Me-Phe)-R8*-Glu-Tyr-Trp-Ala-Leu-Leu-S5*-NH_2_ |
| MP-313 | H-DThr-DAla-DTrp(6-F)-DTyr-DAla-DAsn-DPhe(4-CF3)-DGlu-S5*-DLeu-DLeu-R5*-NH_2_ |
| MP-990 | Ac-Leu-Thr-DPhe-R8*-Glu-Tyr-Trp-Ala-Gln-Leu-S5*-Ala-Ala-Ala-Ala-Ala-DAla-NH_2_ |

* = site of stapling; S5 = (S)-2-(4’-pentenyl) alanine; R8 = (R)-2-(7’octenyl) alanine; Aib = 2-aminoisobutyric acid; mPEG24 = mono methylated polyethylene glycol; E8 = (S)-2-(7’octenyl) alanine; R5 = (R)-2-(4’-pentenyl) alanine; B5 = 2-amino-2-(pent-4-enyl)hept-6-enoic acid; Cba = cyclobutyl alanine; D-amino acids = DAaa
